# Supplementary material for: Transglutaminase 3 crosslinks the secreted gel-forming mucus component Mucin-2 and stabilizes the colonic mucus layer
Source: Nat Commun. 2022 Jan 11;13:45. doi: 10.1038/s41467-021-27743-1 (PMC8752817; doi:10.1038/s41467-021-27743-1)
Supplement: Supplementary file 1 — Supplementary Information [file 41467_2021_27743_MOESM1_ESM.pdf]

Supplementary Information

Transglutaminase 3 crosslinks secreted MUC2 and stabilizes the colonic mucus layer

List of contents

Supplementary Fig. S1: Positive control for the α-TGM2 antibody in the small intestine and specificity analysis of α-TGM2 and α-TGM3 antibodies.

Supplementary Fig. S2: Limit of Detection (LoD) and Limit of Quantification (LoQ) evaluation for transglutaminase activity quantification.

Supplementary Fig. S3: Analysis of the impact of host and microbial proteases on MUC2 degradation.

Supplementary Fig. S4: Mucus growth rate and mucus penetrability analysis of *Tgm3*$^{-/-}$ animals.

Supplementary Fig. S5: Combined Fluorescence in situ hybridization (FISH) and MUC2 staining of colon tissue sections from WT and *Tgm3*$^{-/-}$ mice.

Supplementary Fig. S6: mRNAseq expression data for members of the cathepsin protease family in colonic goblet and non-goblet cells extracted from Nyström *et al.*[1].

Supplementary Reference

Supplementary Figures

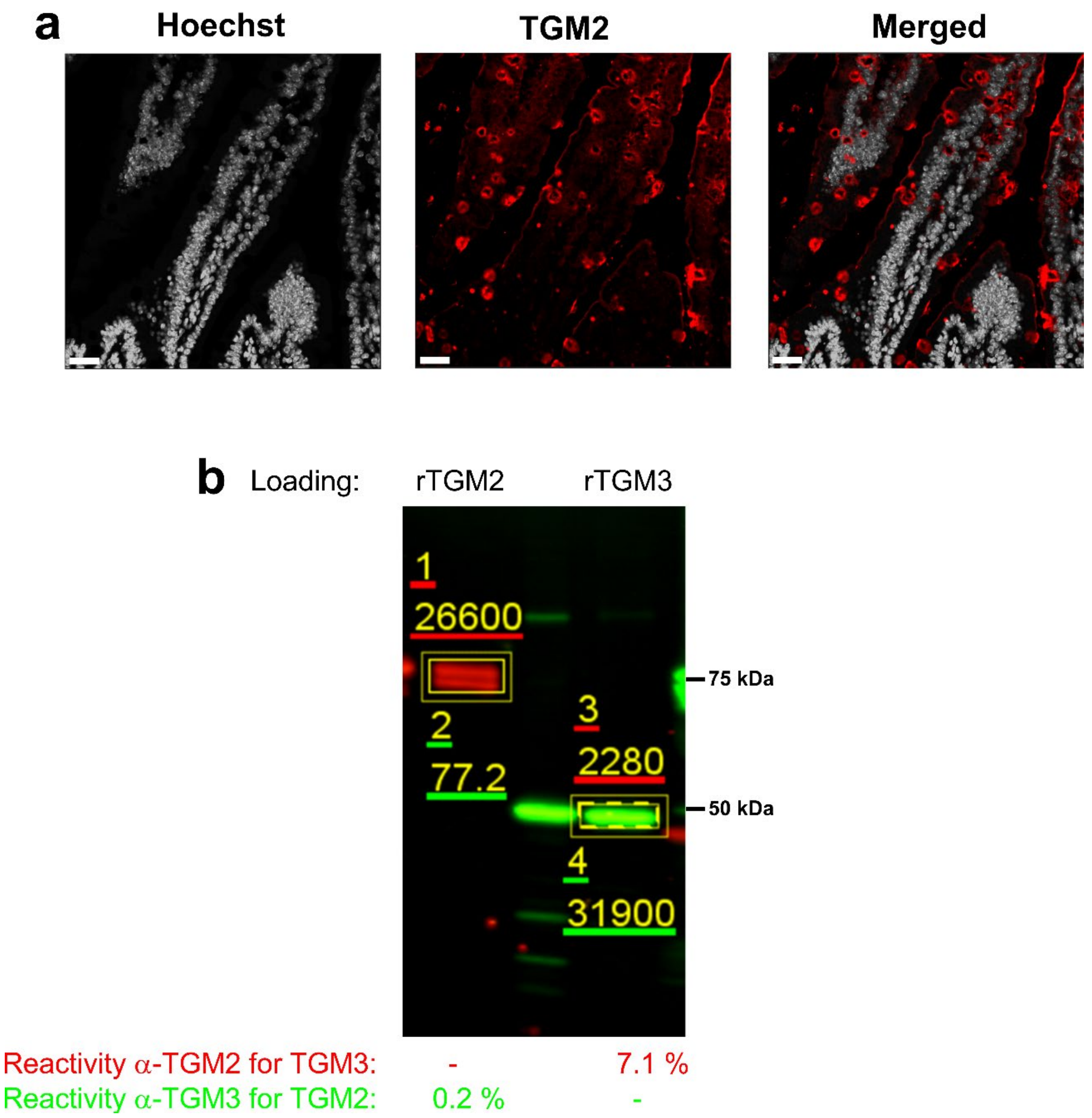

Supplementary Figure S1| Positive control for the α-TGM2 antibody in the small intestine and specificity analysis of α-TGM2 and α-TGM3 antibodies.

(a) Ileal tissue specimen was stained for TGM2 using the CUB7402 antibody (red). Nuclei were counterstained using the Hoechst stain (grey). The staining shows that the antibody detects TGM2 on paraffin-embedded tissue sections. n=3. The scale bar corresponds to 30 μm.

(b) Recombinant TGM2 (rTGM2) and TGM3 (rTGM3) were analysed for their recognition by the α-TGM2 and α-TGM3 antibodies used in this study by the Odyssey Clx workstation analysis software. A cross-reactivity of 0.2 % for the α-TGM3 antibody towards rTGM2 (signals 1=rTGM2 staining by α-TGM2 and 2=TGM2 staining by α-TGM3) and of 7.1 % for the α-TGM2 antibody towards rTGM3 (signals 3=rTGM3 staining by α-TGM2 and 4=rTGM3 staining by α-TGM3) was reveiled based on the signal intensity (n=3).

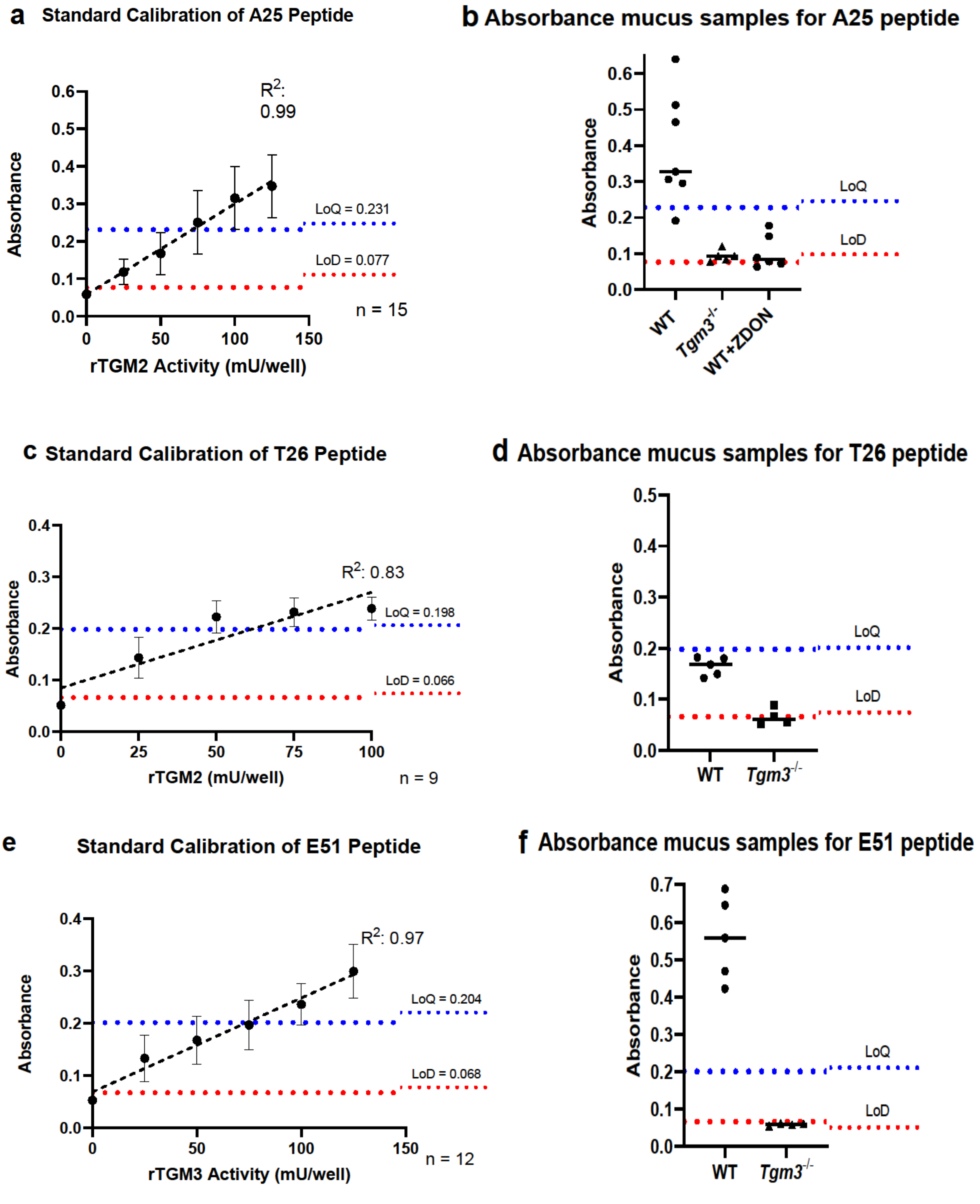

Supplementary Figure S2| Limit of Detection (LoD) and Limit of Quantification (LoQ) evaluation for transglutaminase activity quantification.

LoD and LoQ were evaluated for the biotinylated peptide substrates used in the quantitative assay (a: peptide A25; c: peptide T26 and e: peptide E51). Three to five independently measured calibration curves consisting of three technical replicates were merged. The absorbance values before multiplication with the dilution factor of the mucus samples from the respective mouse strains are shown aside with their calibration curves (b: peptide A25; d: peptide T26; f: peptide E51). The LoD was calculated as the mean absorbance of the used sample buffer plus three-fold of its standard deviation and the LoQ equals three times the LoD.

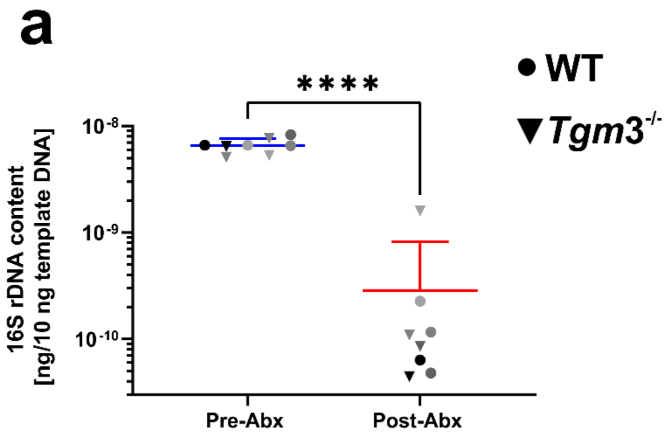

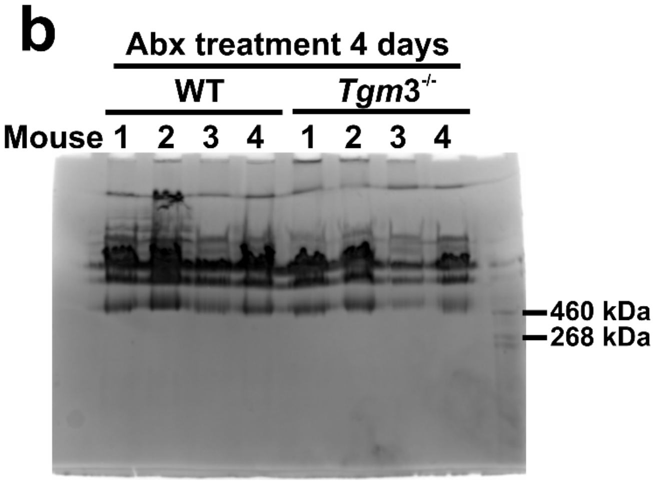

Supplementary Figure S3| Analysis of the impact of host and bacterial proteases on MUC2 degradation.

Female, age-matched WT and *Tgm3*$^{-/-}$ mice were cohoused and treated for 4 days with an antibiotica cocktail (Abx) that was added to the drinking water consisting of ampicillin, neomycin, vancomycin (each 0.1 % (w/v)) as well as metronidazole (0.05 % (w/v)). Fecal pellets were collected before and after the Abx-treatment and the relative bacterial content quantified by qPCR using a pan-16S rDNA primer set against a calibration curve of chromosomal *Escherichia coli* DNA.

(a) Abx-treatment led to an approximately 100-fold reduction of the bacterial load in all mice except one. (n=4 per mouse strain). Circles mark WT and triangels *Tgm3*$^{-/-}$ animals. Corresponding samples from the same animal pre- and post-Abx treatment are marked in the same grey scale. The blue and red bar mark the mean pre- and post-Abx treatement. Data are presented as mean ± standard deviation. A pair-wise, two-tailed t-test was performed ($p<0.0001$).

(b) After Abx-treatment the mice were sacrificed and the mucus extracted from the colon followed by composite agarose-PAGE and staining of MUC2 using Alcian Blue. No difference between WT and *Tgm3*$^{-/-}$ mucus was detected (n=4 per mouse strain).

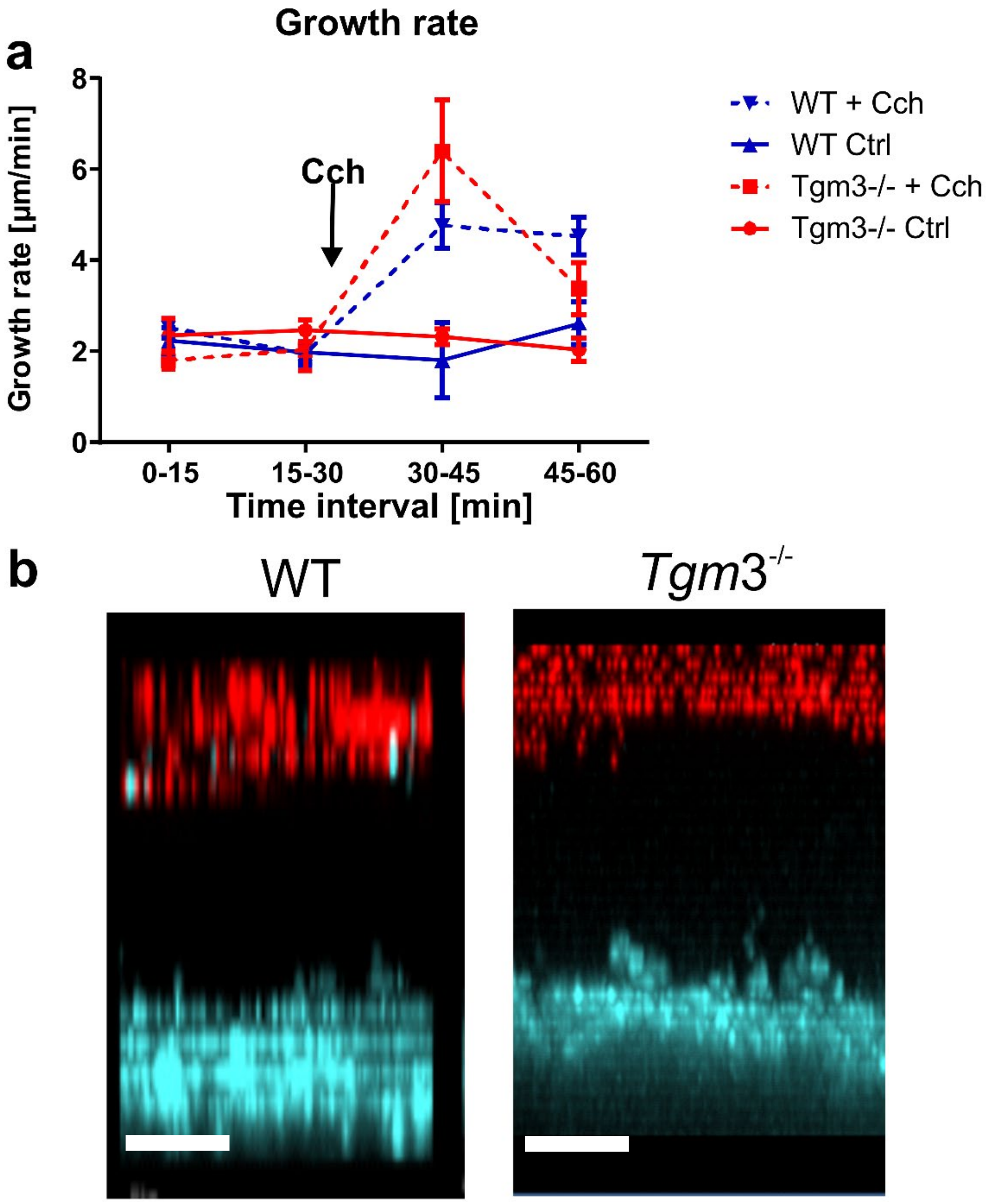

Supplementary Figure S4| No change in mucus growth rate and mucus penetrability in *Tgm3*$^{-/-}$ animals.

(a) The mucus growth rate of WT and *Tgm3*$^{-/-}$ mice was compared with and without carbachoal (Cch) induction using an *ex vivo* approach by mounting the distal colon into a horizontal perfusion chamber. Data are presented as mean ± standard deviation. No significant differences between WT (n=5) and *Tgm3*$^{-/-}$ (n=4) animals were observed.

(b) *Tgm3*$^{-/-}$ mice show a normal non-penetrable mucus layer when fluorescently-labelled beads of 1 µm diameter were placed on top of the mucus layer of mounted colon specimen *ex vivo*. The scale bar corresponds to 50 µm. Five WT animals and four *Tgm3*$^{-/-}$ animals were analysed.

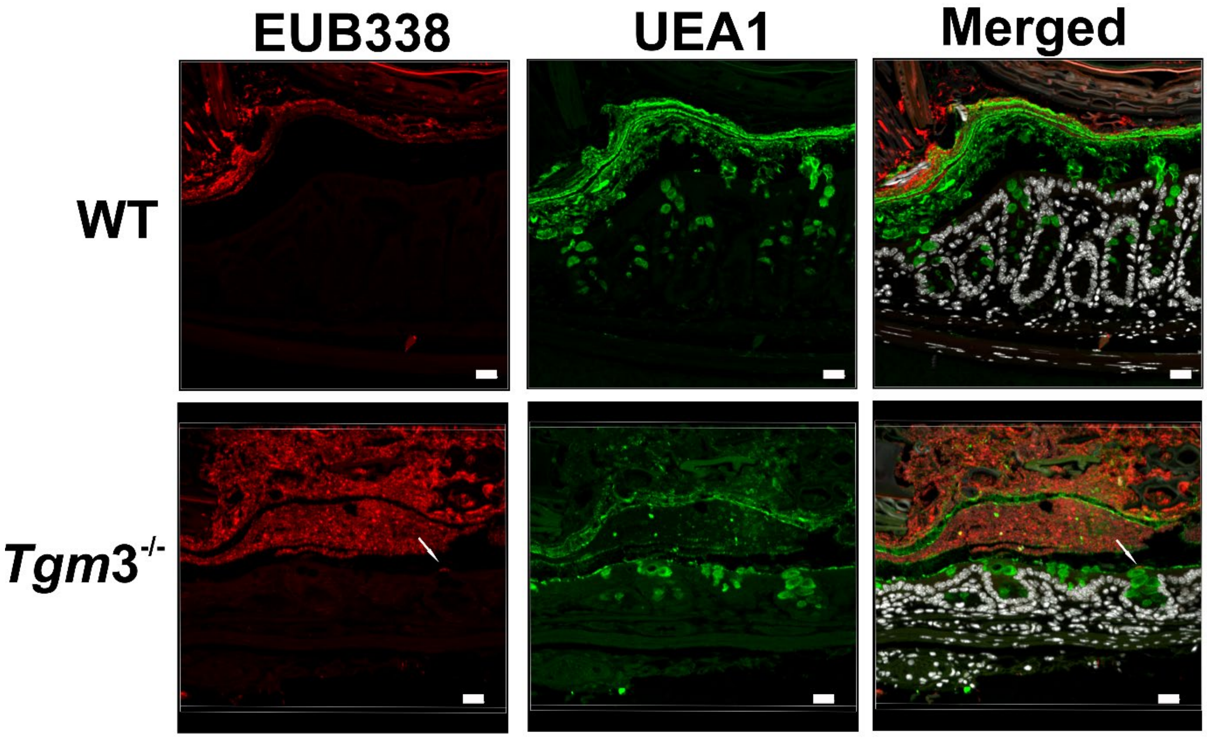

Supplementary Figure S5| Combined Fluoresence *in situ* hybridization (FISH) and MUC2 staining of colon tissue sections.

Carnoy-fixed tissue specimen from WT and TGM3-deficient mice were stained with the Alexa555-labelled pan-bacterial DNA probe EUB338 (red) and the UEA1-lectin for mucus/MUC2 (green). Nuclei were counterstained with the Hoechst dye (grey). The white arrow marks bacteria at the surface of the epithelium. n=3. The white scale bars correspond to 20 µm.

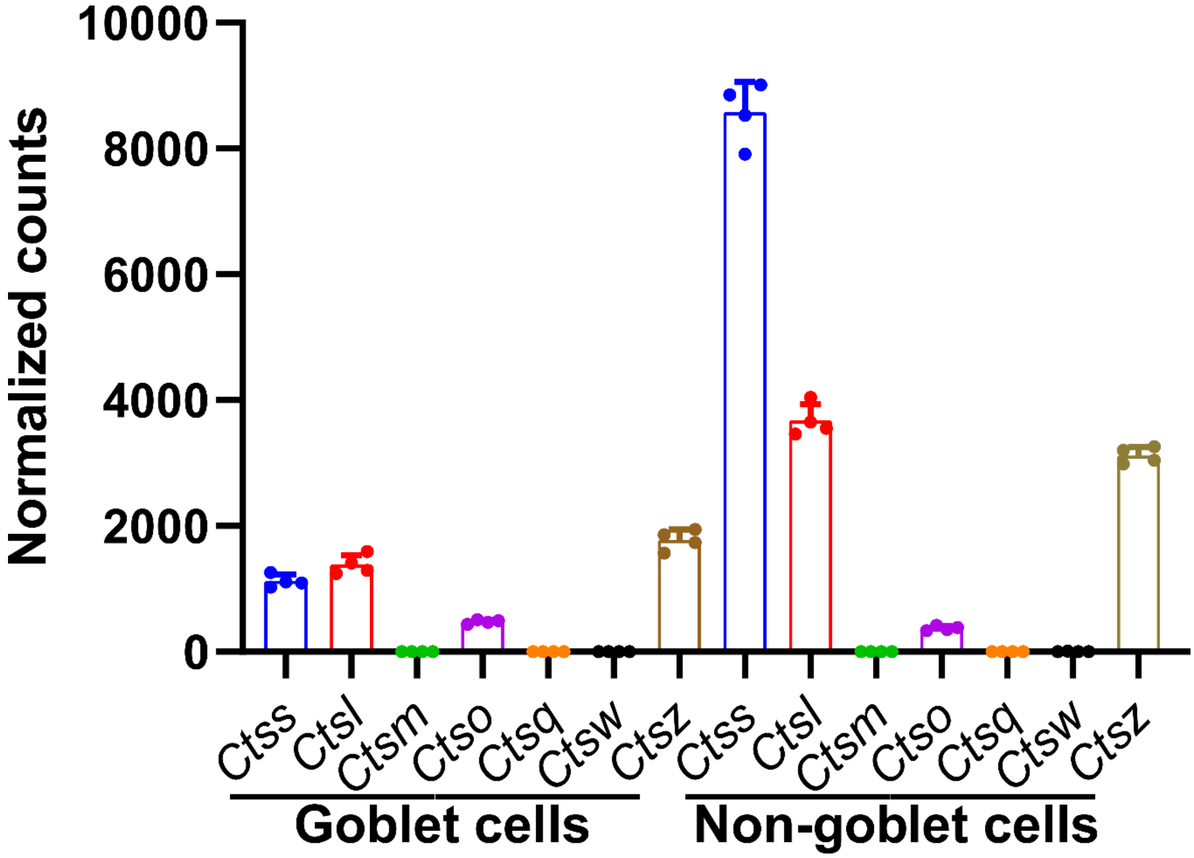

Supplementary Figure S6| mRNAseq expression data for members of the cathepsin protease family in colonic goblet and non-goblet cells extracted from Nystrom *et al.*[1]. Data are presented as mean ± standard deviation. Four biological replicates were analysed.

Supplementary Reference

1. Nystrom, E.E.L. *et al.* An intercrypt subpopulation of goblet cells is essential for colonic mucus barrier function. *Science* **372** (2021).
