# Supplementary material for: Transglutaminase 3 crosslinks the secreted gel-forming mucus component Mucin-2 and stabilizes the colonic mucus layer
Source: Nat Commun. 2022 Jan 11;13:45. doi: 10.1038/s41467-021-27743-1 (PMC8752817; doi:10.1038/s41467-021-27743-1)
Supplement: Supplementary file 3 — Description of Additional Supplementary Files [file 41467_2021_27743_MOESM3_ESM.docx]

Description of Additional Supplementary Files

Title: Supplementary Movie 1

Description: Ex vivo transglutaminase activity analysis. After microdissection colonic tissue was mounted in a perfusion chamber and kept at 37 °C. The transglutaminase substrate E51 (purple) was added together with the UEA1 lectin (green) for counter-staining of the mucus and calcein violet (blue) for visualisation of the tissue surface for 30 minutes. After washing away non-incorporated substrate the E51-incorporation was analysed on a confocal microscope.

Title: Supplementary Movie 2

Description: Ex vivo mucus integrity assay from a WT mouse. After microdissection colonic tissue was mounted in a perfusion chamber that was kept at 37 °C. Pronase was added and fluorescent beads (red) with a diameter of 1 µm were placed on top of the mucus layer. The tissue surface was visualized using Syto 9 (green). A time series over one hour was recorded on a confocal microscope to visualize the respective bead positions.

Title: Supplementary Movie 3

Description: Ex vivo mucus integrity assay from a Tgm3-/- mouse. After microdissection colonic tissue was mounted in a perfusion chamber that was kept at 37 °C. Pronase was added and fluorescent beads (red) with a diameter of 1 µm were placed on top of the mucus layer. The tissue surface was visualized using Syto 9 (green). A time series over one hour was recorded on a confocal microscope to visualize the respective bead positions.
