# Supplementary material for: Transglutaminase 3 crosslinks the secreted gel-forming mucus component Mucin-2 and stabilizes the colonic mucus layer
Source: Nat Commun. 2022 Jan 11;13:45. doi: 10.1038/s41467-021-27743-1 (PMC8752817; doi:10.1038/s41467-021-27743-1)
Supplement: Supplementary file 7 — Reporting Summary [file 41467_2021_27743_MOESM7_ESM.pdf]

nature portfolio

Corresponding author(s): Christian Recktenwald

Last updated by author(s): Nov 25, 2021

Reporting Summary

Nature Portfolio wishes to improve the reproducibility of the work that we publish. This form provides structure for consistency and transparency in reporting. For further information on Nature Portfolio policies, see our Editorial Policies and the Editorial Policy Checklist.

Statistics

For all statistical analyses, confirm that the following items are present in the figure legend, table legend, main text, or Methods section.

n/a	Confirmed	
☐	☒	The exact sample size (*n*) for each experimental group/condition, given as a discrete number and unit of measurement
☐	☒	A statement on whether measurements were taken from distinct samples or whether the same sample was measured repeatedly
☐	☒	The statistical test(s) used AND whether they are one- or two-sided<br>*Only common tests should be described solely by name; describe more complex techniques in the Methods section.*
☐	☒	A description of all covariates tested
☐	☒	A description of any assumptions or corrections, such as tests of normality and adjustment for multiple comparisons
☐	☒	A full description of the statistical parameters including central tendency (e.g. means) or other basic estimates (e.g. regression coefficient) AND variation (e.g. standard deviation) or associated estimates of uncertainty (e.g. confidence intervals)
☐	☒	For null hypothesis testing, the test statistic (e.g. *F*, *t*, *r*) with confidence intervals, effect sizes, degrees of freedom and *P* value noted<br>*Give P values as exact values whenever suitable.*
☒	☐	For Bayesian analysis, information on the choice of priors and Markov chain Monte Carlo settings
☒	☐	For hierarchical and complex designs, identification of the appropriate level for tests and full reporting of outcomes
☒	☐	Estimates of effect sizes (e.g. Cohen's *d*, Pearson's *r*), indicating how they were calculated

Our web collection on statistics for biologists contains articles on many of the points above.

Software and code

Policy information about availability of computer code

Data collection	Graph Pad prism software version 9.0.1. Zen blue (version 3.1) and black (version 2.3) software were used for confocal microscopy. LiCOR Odyssey Clx workstation for Westen Blot analysis. Image lab (version 5.1) for PAGE analysis.
Data analysis	Graph Pad prism was used to draw graphs and for statistical analysis. The MASCOT search engine (version 2.6.0) was used for MS data analysis. The MSconvert software tool (ProteoWizard version 3.0.18146) was used for the generation of *mgf files. For Label-free mass spectrometric quantification of TGM isoforms the MaxQuant software tool (version 1.5.7.4) was used.<br>The StavroX software tool (version 3.6.6) was used for the identification of transglutaminase reaction products. Data processing of immunohistochemistry images was performed using the Imaris software (version 9.5) The Image studio lite software (version 5.2; LiCOR) was used for the analysis of Western Blot data.

For manuscripts utilizing custom algorithms or software that are central to the research but not yet described in published literature, software must be made available to editors and reviewers. We strongly encourage code deposition in a community repository (e.g. GitHub). See the Nature Portfolio guidelines for submitting code & software for further information.

Data

Policy information about availability of data

All manuscripts must include a data availability statement. This statement should provide the following information, where applicable:

- Accession codes, unique identifiers, or web links for publicly available datasets
- A description of any restrictions on data availability
- For clinical datasets or third party data, please ensure that the statement adheres to our policy

The raw data related to Figures 1-4 and Supplementary Figure S3a are provided in the Source data file. The result files of the MASCOT and StavroX searches are also

provided as Source data file. For MASCOT searches the UniProt database (version 06/2017) and an in-house database (http://www.medkem.gu.se/mucinbiology/databases/index.html) containing all human and mouse mucin sequences was used. For the detection of cross-links in MUC2 the UniProt sequence of murine MUC2 (UniProt identifier: Q80Z19) was used. MS raw data from this manuscript are uploaded to the PRIDE proteomics exchange server and have the identifier PXD029071. The proteomics data set for label-free quantification used has been published (Nystrom et al. Science 2021) and deposited to the ProteomeXchange Consortium (http://proteomecentral.proteomexchange.org) with the dataset identifier PXD011527. The bulk RNA-seq data sets (https://www.ncbi.nlm.nih.gov/geo/query/acc.cgi?acc=GSE144363 GSE144363) are deposited in GEO (https://www.ncbi.nlm.nih.gov/geo/) and belong to the superserie GSE144436 and have been published (Nystrom et al. 2021). All other data supporting the findings in this study are available in the manuscript, its Supplementary Information, or from the corresponding author upon reasonable request. The reporting summary of this article is available as Supplementary Information File.

Field-specific reporting

Please select the one below that is the best fit for your research. If you are not sure, read the appropriate sections before making your selection.

[x] Life sciences [ ] Behavioural & social sciences [ ] Ecological, evolutionary & environmental sciences

For a reference copy of the document with all sections, see nature.com/documents/nr-reporting-summary-flat.pdf

Life sciences study design

All studies must disclose on these points even when the disclosure is negative.

Sample size

The sample sizes were determined empirically and no statistical method was used to predetermine them.
For the RNAseq expression data and label-free quantification of TGMs the data from the work of Nystrom et al. Science 2021 were mined and we had therefore no influence on the sample size. For the immunohistochemistry and Western Blot analyses of TGM abundance in the colonic tissue and mucus at least three animals were analysed and as similar results were obtained in these orthogonal methods with individual biological replicates this number was considered sufficient following the 3R guidelines for animal studies.
For the qualitative TGM activity-in vitro studies as well as the ex vivo TGM activity and mucus integrity studies three animals per mouse strain were analysed and as similar results were obtained following the 3R guidelines for animal studies.
For the quantitative in vitro activity studies four Tgm3-/- mice were analysed per glutamine-donor substrate. As all of them showed no detectable and/or quantifiable activity this n was considered as sufficient following the 3R guidelines for animal studies. For the WT strain between five and seven animals were analysed in order to get a more precise enzymatic activity value. This number of animals was considered as sufficient since they passed the Shapiro-Wilk test for normality distribution.
For the analysis of the biochemical alterations of mucus/MUC2 caused by the loss of TGM3 three individual animals per mouse strain were analysed in the respective experiments and as similar results were obtained this number was considered to be sufficient following the 3R guidelines for animal studies.
Sample size for the DSS study was chosen based on those used in similar studies to which we expected similar effect sizes (Petersson J. et al. Am J Physiol Gastrointest Liver Physiol 2011; Johansson M. et al. PlosOne 2010; Xiao F et al. Acta Physiologica 2013).
Sample sizes for experiments in the Supplementary Information were either chosen based on those used in similar studies to which we expected similar effect sizes (Birchenough GMH et al. Science 2016 for Supplementary Fig. S3-S5) or when the validation studies for antibodies and the test for the determination of TGM activity showed that the experimental set up is functional (Supplementary Fig. S1 and S2). The RNAseq data for the expression of cathepsins were mined from the study performed by Nystrom et al. Science 2021 where we had no influence on the sample sizes.
The details of sample sizes can be depicted from Figure legends and the methods section.

Data exclusions

For the colon length comparisons one mouse from the Tgm 3-/- group was excluded as the colon broke into pieces during dissection and could not be put together for a reliable measurement.

Replication

All experimental findings were replicated on at least three individual animals (biological replicates). All attempts at replication were successful.

Randomization

WT and TGM3-deficient mice were sex- and age-matched and co-housed to ensure microbiota normalisation before the animals recieved the same treatment under the same environmental conditions.

Blinding

For biochemical and immunohistochemical experiments blinding of the investigators was not relevant as the collected samples were treated equally and the data analyses were performed under the same conditions. For DSS challenge experiments, age- and sex-matched mice from both genotypes were co-housed prior to and during the experiment. The measurements of all objective disease activity index paramenter were performed concurrently. Therefore blinding was not practical due to limited number of staff available and as investigators needed to distinguish the individual animals from the same cage. Investigators were blinded for subsequent analysis of collected tissues from DSS experiments, e. g. histochemical analysis.

Reporting for specific materials, systems and methods

We require information from authors about some types of materials, experimental systems and methods used in many studies. Here, indicate whether each material, system or method listed is relevant to your study. If you are not sure if a list item applies to your research, read the appropriate section before selecting a response.

Materials & experimental systems

n/a	Involved in the study	
	x	Antibodies
x		Eukaryotic cell lines
x		Palaeontology and archaeology
	x	Animals and other organisms
x		Human research participants
x		Clinical data
x		Dual use research of concern

Methods

n/a	Involved in the study	
x		ChIP-seq
x		Flow cytometry
x		MRI-based neuroimaging

Antibodies

Antibodies used

Primary antibodies:
Transglutaminase 3/TGM3 Antibody (Novus Biologicals #NBP1-57678)
TGM2 Monoclonal antibody (ThermoFisher #MA5-12739, CUB7402)
MUC2 antibody [C3] (Genetex #GTX100664)

Secondary Antibodies:
Western Blot:
Goat-anti-mouse-IgG1 coupled to IR680LT dye (LI-COR #926-68050 Lot no.: 80220-05)
Goat-anti-rabbit-IgG coupled to AlexaFluor 790 (ThermoFisher #A11369 Lot no.: 1854591)

Immunohistochemistry:
Goat-anti-mouse-IgG coupled to AlexaFluor647 (ThermoFisher #A21236 Lot no.: 1654338)
Goat-anti-rabbit-IgG coupled to AlexaFluor 647 (ThermoFisher #A21245 Lot no.: 1660844)

Validation

The TGM3 antibody (NBP1-57678) has been validated in this study by the use of sections from WT and Tgm3-/- animals for IHC and by recombinantly expressed TGM3 for Western Blot analyses as well as by the manufacturer.
The TGM2 antibody has been validated in this study by the use of small intestinal tissue sections for IHC and colonic tissue sections after DSS treatment and by recombinantly expressed TGM2 for Western Blot analyses as well as by the manufacturer.
Cross-reactivity between the two antibodies was evaluated in Supplementary Figure S1b.
Orthogonal validation for the MUC2 antibody was performed by the manufacturer and can be found on the manufacturer`s website together with relevant citations.

Animals and other organisms

Policy information about studies involving animals; ARRIVE guidelines recommended for reporting animal research

Laboratory animals

Mus musculus; C57/BL6; C57/BL6-Tgm2-/- and C57/BL6-Tgm3-/-; male and female; 8-15 weeks

Wild animals

The study did not involve wild animals.

Field-collected samples

The study did not involve samples collected in the field.

Ethics oversight

Animal protocols were performed in agreement to the Swedish legislation: Jordbruksverket (Sweden). Ethical permits: 2285/19 and 2292/19.

Note that full information on the approval of the study protocol must also be provided in the manuscript.
